# The mediating role of anxiety and depression between problematic social media use and bulimia nervosa among Lebanese university students

**DOI:** 10.1186/s40337-023-00776-1

**Published:** 2023-03-29

**Authors:** Michel Sfeir, Clara Rahme, Sahar Obeid, Souheil Hallit

**Affiliations:** 1 Department of Clinical Psychology, University of Mons, Mons, Belgium; 2 Institute of Psychology (IP), Faculty of Social and Political Sciences, University of Lausanne, Lausanne, Switzerland; 3 Research Department, Psychiatric Hospital of the Cross, Jal Eddib, Lebanon; 4 Social and Education Sciences Department, School of Arts and Sciences, Lebanese American University, Jbeil, Lebanon; 5 School of Medicine and Medical Sciences, Holy Spirit University of Kaslik, P.O. Box 446, Jounieh, Lebanon; 6 Applied Science Research Center, Applied Science Private University, Amman, Jordan

**Keywords:** Bulimia nervosa, Anxiety, Depression, Problematic social media use, Lebanon

## Abstract

**Background:**

Bulimia nervosa (BN) is a disorder that is characterized by binge eating and inappropriate compensatory behavior to control weight. The aim of this study was to evaluate the mediating role of anxiety and depression between problematic social media use (PSMU) and BN among a sample of Lebanese university students.

**Methods:**

This cross-sectional study was carried out between July and September 2021; a total of 363 university students was recruited through convenience sampling. The PROCESS SPSS Macro version 3.4, model four was used to test the indirect effect and calculate three pathways. Pathway A determined the regression coefficient for the effect of PSMU on mental health issues (depression/anxiety); Pathway B examined the association between mental health issues on BN, and Pathway C’ estimated the direct effect of PSMU on BN. Pathway AB was used to calculate the indirect effect of PSMU on BN via depression/anxiety.

**Results:**

Results showed that depression and anxiety partially mediated the association between PSMU and BN. Higher levels of PSMU were associated with more depression and anxiety; higher depression and anxiety were associated with more BN. PSMU was directly and significantly associated with more BN. When entering anxiety (M1) then depression (M2) as consecutive mediators in a first model, the results showed that only depression mediated the association between PSMU and bulimia. When taking depression (M1) then anxiety (M2) as consecutive mediators in a second model, the results showed that the mediation PSMU → Depression → Anxiety → Bulimia was significant. Higher PSMU was significantly associated with more depression, which was significantly associated with more anxiety, which was significantly associated with more bulimia. Finally, higher PSMU was directly and significantly associated with more bulimia

**Conclusion:**

The current paper highlights the relationship that social media use has on BN and other aspects of mental health such as anxiety and depression in Lebanon. Future studies should replicate the mediation analysis conducted in the current study while taking into account other eating disorders. Additional investigations of BN and its correlates must strive to improve the comprehension of these associations’ pathways through designs that allow to draw temporal frameworks, in order to efficiently treat this eating disorder and prevent its negative outcomes.

## Abstract (plainenglishlanguage)

Bulimia nervosa, an eating disorder, is characterized by an impulsive consumption of food in a short period of time, followed by behaviors that compensate the eating such as vomiting or excessive exercise in order to avoid weight gain. Individuals with problematic social media use were found to have higher levels of bulimia symptoms. Symptoms of bulimia can also be associated with both depression and anxiety. The aim of the current study was to examine the mediating role of anxiety and depression between problematic social media use and bulimia nervosa. The results of our study found that problematic social media use was directly associated with more bulimia nervosa and also associated with higher depression and anxiety, both of which were associated with bulimia nervosa. Tackling associated disorders may help reduce symptoms of bulimia nervosa. Clinicians should carefully examine these associations while assessing and implementing treatment plans.

## Introduction

The most severe mental illnesses affecting adolescents and young adults today are eating disorders (EDs), such as anorexia nervosa (AN), bulimia nervosa (BN), and binge eating disorder (BED) [1]. Worldwide, both males and females of all ages exhibit BN, which is linked to increased mortality risk [2]. BN is defined as “recurrent binge eating episodes along with inappropriate compensatory behaviors, and is linked to serious medical problems, mental comorbidity, and psychosocial impairment” [3]. People with BN may exhibit bursts of impulsive consumption of a lot of food in a short amount of time, followed by compensatory behaviors (such as excessive exercise, vomiting, laxative abuse, limited food intake) to prevent weight gain [4, 5]. In the systematic review of Galmiche et al., the lifetime prevalence rates of BN ranged from 0.1% to 1.3% in men and from 0.3% to 4.6% in women [6]. The peak age of incidence of BN ranged between 15 and 29 years [2]. In addition, compared to persons without an ED, all participants with EDs had greater median incomes and lower education [7]. A prior study conducted on EDs in Lebanon found that BN was the most prevalent ED (46.1%) ,followed by anorexia nervosa (39.4%) and binge eating (14.4%) [1]. Additionally, prior research revealed that 11.4% of university students in Lebanon had AN, BN, or BED diagnosis and that 21.2% were at risk for developing an ED [8]. ED behaviors are thought to be painful, as people are often engaged in extreme measures to alter body shape and abate concern about the body [9].

Body dissatisfaction is often used as a term to describe the body-related negative self-evaluation of an individual [10]. Body dissatisfaction was a strong prospective predictor of the severity of suicidal thoughts, and BN symptoms (binge eating and purging) predicted suicidal ideation [9]. When attempting to understand the emergence of body dissatisfaction and EDs, culture is a crucial factor to take into account since it determines the environment in which attitudes regarding the body are formed [11]. Lebanese media, similar to Western cultures, promotes the culture of ‘‘thinness’’ and ‘‘perfection’’ [12]. Lebanese are more susceptible to media messages encouraging them to eat less and exercise more in order to lose weight or gain muscle mass [13–18]. They also fear social criticism and are more susceptible to peer opinions. In addition, compared to their Cypriot peers, Lebanese women are more self-conscious about their body size [13].

EDs are multi-factorial and include biological, psychological, intrapersonal, and environmental influences. Exposure to media, one environmental factor, has been linked to the emergence of these problems and is probably mediated by thin-ideal internalization [19]. According to the biopsychosocial model, problematic social media use (PMSU) is characterized by the presence of addiction-like symptoms such as mood modifications (i.e., alterations in mood states with the excessive social media use), tolerance (i.e., an increase in the amount of time spent on social media), withdrawal symptoms (i.e., feeling contradicted or irritable when restricted from using social media), conflict (i.e., relationship problems as a result from using social media) and relapse (i.e., going back to social media use after stopping for a while) [20, 21]. Scales to assess Social Media Use as a type of addiction include several criteria of behavioral addictions such as: preoccupation, tolerance, withdrawal, persistence, displacement, problem, deception, escape and conflict [22, 23]. A previous research found a significant positive correlation between BN and the time spent on social networking sites [24]. Furthermore, in two recent meta-analyses, Hinojo-Lucena and colleagues found that those with problematic use of internet had significantly higher rates of both EDs (AN, BN, and BED) and ED-related symptoms (food obsession, loss of control eating, and dieting) [25]. While aiming to examine an association between social media use and eating concerns, a study found a strong association between the two [19]. Social media is more widely available at young age; just one click can set off a range of ideas and behaviors that mimic EDs in those people, to conform to what society considers to be attractive [19]. With that being said, the pressure of media influence was associated with more EDs (restrained and emotional eating) among Lebanese undergraduates [26].

While many factors contribute to the development of BN, having a comorbid disorder can be associated with more severe symptoms of EDs [27, 28]. Godart and colleagues (2000) found that anxiety disorders were frequently present before the occurrence of EDs [29]. The results of a previous study suggested that the comorbidity of an ED with anxiety and depression was high [30]. An earlier network analysis study revealed that the anxiety symptoms of shakiness, unsteadiness, and dizziness were very central and closely related to the BN symptoms in the anxiety and BN network. Similarly, in the depression and BN network, the lack of interest in sex and changes in appetite were highly central [31]. Therefore, by identifying the core symptoms of the comorbid disorders (e.g., comorbid anxiety and depression symptoms), treatment of BN could be improved to concentrate on these symptoms. Anxiety positively contributed to addictive social networking, with social media use shown to be positively associated with depression among young adults [32, 33]. Depressive symptoms were also found to predict eating behaviors ten years later [34, 35]. A descriptive review found that the levels of neurocognitive alterations and impairment in individuals with AN were proportional to the severity of depressive symptoms [36]. It is noteworthy to also mention that depression can be secondary to EDs according to the results of a longitudinal study [37]. Similarly, anxiety moderated the association between body dissatisfaction and restrained eating; when levels of anxiety are high, body image dissatisfaction was more strongly associated with restrained eating [35]. Furthermore, depression moderated the association between body dissatisfaction and orthorexia nervosa [38].

As EDs are very uncommon in the general population, help seeking is frequently avoided or put off for many reasons, such as denial (especially in the case of AN) or stigma and shame (especially in the case of BN) [2]. Most of the epidemiological research on EDs has been conducted in Western nations. There is evidence to support the idea that non-Western nations are not immune to EDs where EDs are spreading, especially in the Middle East [1]. Mental health issues are frequently underestimated in developing countries, although they were shown to be prevalent in Lebanon following the COVID-19 pandemic [39], particularly in the context of a severe socio-economic crisis and political instability [40, 41]. Moreover, Arab cultures and mentalities favor and work hard for a thin and toned body, which puts a lot of pressure on people, therefore, emphasizing the importance of studying BN in these populations. In fact, the sociocultural changes in the Arab countries have led to a shift from the admiration of curvy bodies to thin ones, a goal achieved by following ED behaviors [35, 42]. In view of the lack of previous studies that assess the correlates of BN in Lebanon, the aim of this study was to evaluate the mediating effect of anxiety and depression between PSMU and BN in a sample of Lebanese university students. We hypothesize that depression and anxiety may mediate the association between BN and PSMU, where an increase level of PSMU would be associated with higher levels of depression and anxiety, which would be associated with higher BN.

## Methods

### Study design and participants

This cross-sectional study was carried out between July and September 2021. A total of 363 university students was recruited through convenience sampling from several universities in Lebanon's governorates. Involved people were encouraged to visit a website that would guide them to the consent form, information form (purpose of the current study, anonymity, voluntariness of consent to research), and questionnaire. The data was collected online using the snowball technique in order to reach the target number. All participants responded willingly to the survey. There were no fees for participating in the study. All university students over the age of 18 were eligible to participate. Excluded were only those who refused to complete the survey and those who were not university students; no other exclusion criteria were applied [43].

### Minimal sample size calculation

According to the G-power, a minimum of 316 students was deemed necessary to have enough statistical power, based on a 5% risk of error, 80% power, f^2^ = 2.5% and 10 factors to be entered in the multivariable analysis.

### Questionnaire and variables

The Arabic self-administered questionnaire with closed-ended questions was anonymous; the questionnaire required approximately 20 minutes to be completed. The questionnaire consisted of different sections. The first part clarified socio-demographic characteristics: age, sex, marital status, and household crowding index. The latter, reflecting the socioeconomic status of the family, was calculated by dividing the number of persons in the house by the number of rooms in the house excluding the bathrooms and kitchen [44]. The physical activity index was calculated by multiplying the intensity by the frequency by the time of physical activity [45].

The second part of the questionnaire included the following scales:

#### Eating attitude test (EAT-26)

The EAT, validated in Lebanon in Arabic [46, 47], was used to assess disordered food attitude. The questionnaire comprises twenty-six questions each with six response options, varying from infrequently/almost never/never (0) to always [3]. It is divided into three subscales: dieting (avoidance of fatty foods and preoccupation with thinness), bulimia and food preoccupation, and oral control (self-control over food and social pressure to gain weight). The total score was calculated by summing all questions answers and can vary from 0 to 78. A score of 20 or above indicates possible disordered food attitudes. In this study, only the bulimia subscale was used. The bulimia scale included items such as: “I vomit after I have eaten”. The Cronbach’s alpha in this study was 0.87.

#### Body dissatisfaction subscale of the eating disorder inventory‑second version (EDI‑2)

The body dissatisfaction subscale evaluates the degree of dissatisfaction to the overall body, and to particular body element. It is made of nine items (i.e., “I am satisfied with the shape of my body”), scored on a 4-point Likert scale, from never (0) to always [3]. Higher scores correspond to a higher level of body dissatisfaction [48]. The Arabic version of the scale was used in a previous study [49]. The Cronbach’s alpha in this study was 0.60.

#### Social media disorder scale (SMD)

Validated in Lebanon in Arabic [50], the short form of the SMD was used in this study. It is composed of 9 items (i.e. “Over the last year, have you often felt bad when you when you could not use social media?”), with higher scores reflecting more problematic social media use [22]. The Cronbach’s alpha in this study was 0.79.

#### Lebanese anxiety scale (LAS-10)

Lebanese Anxiety Scale (LAS-10) is a 10-item instrument in Arabic measuring the severity of anxiety symptoms among Lebanese adults [49] and adolescents [50]. This scale was previously used in Lebanon [51, 40]. In LAS-10, the first seven questions are graded from 1 to 10, and the last three questions are graded from 1 to 4 based on the repetitive manifestation of symptoms (i.e., “I feel that the difficulties are accumulating to the point where I can’t get through them”). Higher scores indicate higher anxiety levels. The Cronbach’s alpha in this study was 0.89.

#### Patient health questionnaire (PHQ-9)

The PHQ-9 is a 9-item self-report scale (i.e. “Over the past two weeks, how often have you been bothered by the following: little interest or pleasure in doing things”), previously validated in Lebanon in Arabic [52], which is used to assess and check the severity of depression. PHQ-9 total score ranges from 0 to 27, with a cut-off point of 0–4 indicates no depressive symptoms, 5–9 mild depressive symptoms, 10–14 moderate depressive symptoms, 15–19 moderately-severe depressive symptoms, and 20–27 severe depressive symptoms [53]. The Cronbach’s alpha in this study was 0.90.

### Statistical analysis

SPSS software version 25 was used to conduct data analysis. The normality of the BN score was verified via the skewness and kurtosis values varying between -1 and +1 [54]. A bivariate analysis using the Pearson correlation test served to assess the relationship between the BN score and other continuous variables, whereas the Student t test was used to compare two means. A linear regression was conducted taking the BN score as the dependent variable. The PROCESS SPSS Macro version 3.4, model four [42] was used to test the indirect effect and calculate three pathways. Pathway A determined the regression coefficient for the effect of PSMU on mental health issues (depression/anxiety); Pathway B examined the association between mental health issues on BN, and Pathway C’ estimated the direct effect of PSMU on BN. Pathway AB was used to calculate the indirect effect of PSMU on BN via depression/anxiety. A serial mediation analysis was conducted afterwards to test the mediating effect of depression and anxiety consecutively in one model. An indirect effect was deemed significant if the bootstrapped 95% confidence intervals of the indirect pathway AB did not pass by zero [42]. The linear regression and moderation analysis were adjusted over all variables that showed a p<0.25 in the bivariate analysis. Significance was defined at p<0.05.

## Results

### Sociodemographic and other characteristics of the participants

A total of 363 students participated in this study; their mean age was 22.65 ± 3.48 years (min = 18; max = 37), with 61.7% females. The mean BN score was 3.10 ± 4.29. Other characteristics are summarized in Table 1. Moreover, 122 (33.6%) of the participants had eating disorders (EAT scores of 20 or more).

**Table 1:** Sociodemographic and other characteristics of the participants (N = 363)

Variable	N (%)
*Sex*	
Male	139 (38.3%)
Female	224 (61.7%)
*Marital status*	
Single	343 (94.5%)
Married	20 (5.5%)
	Mean ± SD
Age (in years)	22.65 ± 3.48
Body mass index (kg/m^2^)	23.62 ± 4.13
Physical activity index	27.94 ± 20.44
Household crowding index (person/room)	1.01 ± 0.53

SD = Standard Deviation

### Bivariate analysis

The bivariate analysis results are summarized in Tables 2 and 3. Older age (r = − 0.11) was significantly associated with less BN, whereas higher PSMU (r = 0.31), higher body dissatisfaction (r = 0.16), higher anxiety (r = 0.48) and higher depression (r = 0.36) were significantly associated with more BN.

**Table 2:** Bivariate analysis of the categorical variables associated with bulimia.

Variable	Bulimia			
	Mean ± SD	*p*	Effect size	Statistical test used
*Sex*		0.223	0.130	Student t test
Male	2.76 ± 3.92			
Female	3.31 ± 4.50			
*Marital status*		0.833	0.052	Student t test
Single	3.11 ± 4.32			
Married	2.90 ± 3.71			

Numbers refer to mean ± SD

**Table 3:** Correlation matrix of continuous variables.

	1	2	3	4	5	6	7	8	9
1. Bulimia	1								
2. Age	−0.11*	1							
3. Body Mass Index	0.07	0.29***	1						
4. Physical activity index	0.07	−0.13*	−0.08	1					
5. Household crowding index	0.02	−0.12*	0.02	0.02	1				
6. Problematic Social media use	0.31***	−0.18***	−0.06	0.001	0.07	1			
7. Body dissatisfaction	0.16**	0.08	0.07	0.03	0.01	0.17**	1		
8. Anxiety	0.48***	−0.19***	−0.05	0.10	0.06	0.32***	0.13*	1	
9. Depression	0.36**	−0.16**	−0.02	0.03	0.12*	0.32***	−0.07	0.69***	1

*p <.05; **p <.01; ***p <.001; numbers refer to Pearson correlation coefficients.

### Multivariable analysis

A linear regression taking BN as the dependent variable, showed that higher PSMU (Beta = .26), higher anxiety (Beta = .16) and higher body dissatisfaction (Beta = .18) were significantly associated with more BN (Table 4).

**Table 4:** Multivariable analysis: Linear regression (using the ENTER model) taking bulimia as the dependent variable.

	Beta	β	*p*	95% CI
Age	0.01	0.01	0.867	−0.10; 0.12
Body Mass Index	−0.03	−0.03	0.536	−0.14; 0.07
Physical activity index	0.01	0.05	0.246	−0.01; 0.03
Body dissatisfaction	0.18	0.32	**< 0.001**	0.12; 0.24
Problematic Social media use	0.26	0.14	**0.002**	0.10; 0.43
Anxiety	0.16	0.31	**< 0.001**	0.10; 0.23
Depression	−0.01	−0.02	0.754	−0.10; 0.07

*Reference group; Beta = unstandardized beta; β = standardized beta; CI = Confidence interval; numbers in bold indicate significant *p*-values. Nagelkerke R^2^ = .337

### Mediation analysis

**Fig. 1:**
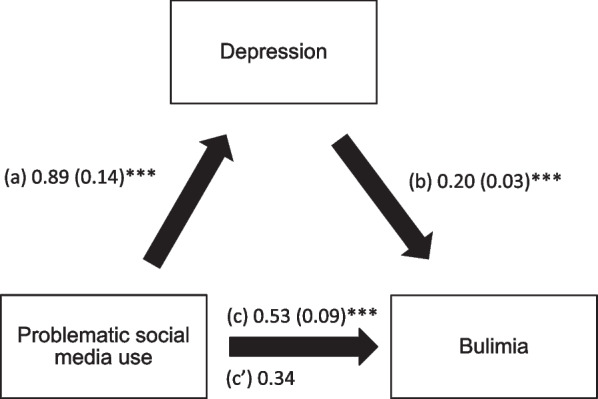
**a** Relation between problematic social media use and depression (R2 = .133); **b** Relation between depression and BN (R2 = .209); **c** Total effect of problematic social media use and BN (R2 = .130); **c’** Direct effect of problematic social media use and BN. Numbers are displayed as regression coefficients (standard error). ***p < 0.001

**Fig. 2:**
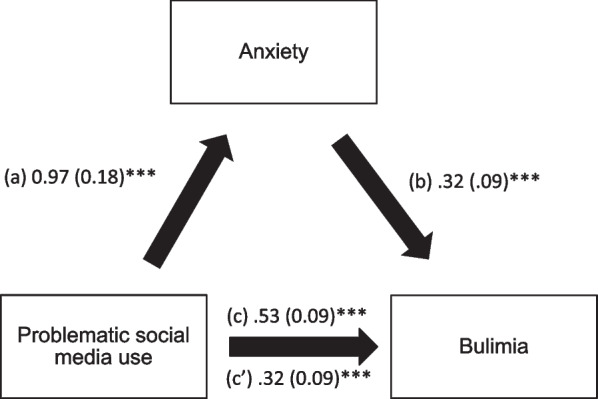
**a** Relation between problematic social media use and anxiety (R2 = .132); **b** Relation between anxiety and BN (R2 = .279); **c** Total effect of problematic social media use and BN (R2 = .130); **c’** Direct effect of problematic social media use and BN. Numbers are displayed as regression coefficients (standard error). **p < 0.01; ***p < 0.001

The results of the mediation analysis (adjusted over age, sex, BMI, physical activity, body dissatisfaction) showed that depression and anxiety partially mediated the association between problematic social media use and BN (Table 5). Higher problematic social media use was significantly associated with more depression/anxiety, whereas more depression/anxiety was significantly associated with more BN. Finally, higher problematic social media use was directly and significantly associated with more BN (Figs. 1 and 2).

**Table 5:** Mediation analyses results, taking problematic social media use as the independent variable, depression/anxiety as the mediators and bulimia as the dependent variable

Mediator	Direct effect			Indirect effect		
	Beta	SE	*P*	Beta	Boot SE	Boot CI
Depression	0.34	0.09	<0.001	0.18	0.05	0.10; 0.29*
Anxiety	0.32	0.09	<0.001	0.21	0.05	0.13; 0.31*

*Indicates significant mediation

### Serial mediation

The mediation analyses were conducted following the indirect effect key below:

Indirect effect 1: PSMU → Depression → Bulimia

Indirect effect 2: PSMU → Anxiety → Bulimia

Indirect effect 3: PSMU → Depression → Anxiety → Bulimia

**Fig. 3:**
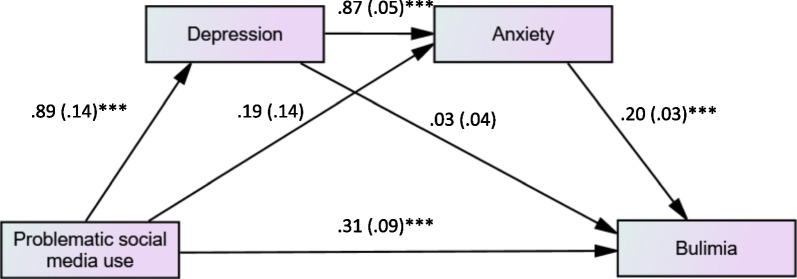
Serial mediation of the effect of problematic social media use on bulimia, taking depression and anxiety as consecutive mediators; ***p < 0.001

The results of the mediation analysis (adjusted over age, sex, BMI, physical activity, body dissatisfaction) showed that depression and anxiety mediated the association between problematic social media use and BN (Table 6). When entering anxiety (M1) then depression (M2) as consecutive mediators in Model 1, the results showed that only depression mediated the association between PSMU and bulimia. When taking depression (M1) then anxiety (M2) as consecutive mediators, the results showed that the mediation PSMU → Depression → Anxiety → Bulimia was significant. Higher PSMU was significantly associated with more depression, which was significantly associated with more anxiety, which was significantly associated with more bulimia. Finally, higher PSMU was directly and significantly associated with more bulimia (Fig. 3).

**Table 6:** Indirect effect analyses results, taking problematic social media use as the independent variable, depression and anxiety as consecutive mediators and bulimia as the dependent variable

	Direct effect			Indirect effect		
	Beta	SE	*p*	Beta	Boot SE	Boot CI
*Model 1: anxiety then depression as consecutive mediators.*						
Total	0.31	0.09	<0.001	0.22	0.05	0.13; 0.33*
Indirect effect 1				0.20	0.05	0.10; 0.31*
Indirect effect 2				0.01	0.02	−0.03; 0.07
Indirect effect 3				0.01	0.03	−0.04; 0.07
*Model 2: depression then anxiety as consecutive mediators*						
Total	0.31	0.09	<0.001	0.22	0.05	0.13; 0.33*
Indirect effect 1				0.02	0.05	−0.07; 0.13
Indirect effect 2				0.04	0.04	−0.03; 0.12
Indirect effect 3				0.16	0.04	0.09; 0.25*

*Indicates significant mediation.

## Discussion

The aim of the current study was to examine the mediating role of depression and anxiety between PSMU and BN among a sample of Lebanese university students. Higher levels of PSMU, anxiety and body dissatisfaction were all correlated with BN. Depression and anxiety partially mediated the association between problematic social media use and BN. When entering anxiety then depression as consecutive mediators, the results showed that only depression mediated the association between PSMU and bulimia. When taking depression then anxiety as consecutive mediators, the results showed that the mediation PSMU → Depression → Anxiety → Bulimia was significant.

### PSMU, depression and anxiety

In a study conducted on 456 Lebanese residents, 107 (23.7%) were classified as having a social media use disorder [55]. The time spent on smartphone screens increased during the COVID-19 pandemic and lockdowns [56] and was associated with more insomnia [57]. The fear of COVID-19 and the lockdown’s impact were both associated with lower general wellbeing, anxiety and depression among Lebanese samples [58, 59]. Results of our study showed that higher PSMU was significantly associated with depression and anxiety, in line with previous findings [60–62]. These authors speculated that the reason for this association may be due to the fact that individuals who engage in online-activities in an excessive way, may neglect healthy aspects of their lives, which could contribute to depressive symptoms. Hence, excessive internet users may more be likely to replace their real-life interactions by online sites than the normal users. They were also found to have more depressive symptoms [61, 62]. A study conducted on Lebanese university students had found a significant association between potential internet addiction and insomnia, depression, anxiety and stress [63]. Moreover, a systematic review of 159 articles, found a bidirectional relationship between PSMU and depression and anxiety; depressed or anxious people may have a higher use of social media, whereas those using social media intensely or excessively may report greater depression or anxiety. The authors of this systematic review concluded that depression and anxiety can be both the causes and consequences of PSMU [64]. In an attempt to evaluate the association between PSMU and its correlates, a Lebanese study found an association between PSMU and anxiety and social phobia [65], in agreement with other international studies [66, 67]. PSMU was associated with an increased level of loneliness, where individuals presenting depressive symptoms may be more prone to use social media rather than face-to-face interactions [68–70]. Adolescents who spend less time in front of their screen and engage in more physical activities were found to have lower risks of reporting mental health problems [71].

### Depression, anxiety and BN

Higher depression and anxiety were both associated with higher BN in the current study, consistent with the findings of a previous study [31]. These authors have found that dizziness, unsteadiness, alterations in appetite and lack of sex were central in BN. Furthermore, 65% of women presenting for treatment of an ED also met the criteria for at least one comorbid anxiety disorder [30]. Previous authors demonstrated that, in addition to distorted body-related thoughts, maladaptive self-evaluative perfectionism - which has been linked to core components of social anxiety disorder – mediated the relationship between bulimic symptoms and social interaction anxiety and fear of public scrutiny, two significant components of social anxiety disorder [72]. As hypothesized by Mitchell and colleagues, the two most common comorbid disorders in BN, generalized and social anxiety, could lead patients with BN to develop an interest in their body weight and shape [73]. Individuals presenting depressive symptoms showed significantly higher symptoms of BN than those without a diagnosis of depression [74]. Improving adherence to and results of ED interventions remain significant priorities for patients with comorbid anxiety disorders as they typically have worse illness courses and outcomes [75]. One symptom that was found to bridge the association between depression, anxiety and BN was physical sensation, which explains how these three disorders may interact [31].

### PSMU and BN

Our study results revealed that higher PSMU was directly associated with more BN, corroborating the results of a previous study [19]. One reason for the study's findings is that people who use social media more frequently are exposed to more pictures and messages that increase the chances of developing ED. The posting and viewing of images and videos are particularly prevalent on some social media platforms, including Instagram, Snapchat, Pinterest, and Tumblr [16, 19]. In Lebanon, the number of social media users at the start of 2022 was equivalent to 75.2% of the total population [76]. Users of social media platforms could be exposed to powerful visual content, such as images that might support the slender ideal [19]. On top of that, it is believed that Western media content and exposure has been shown to significantly affect body image and eating behavior by promoting a ‘‘culture of thinness’’, predicting disordered eating symptoms, body dissatisfaction and a drive to thinness in women [77]. This exposure to thin-ideal images culture was positively associated with body dissatisfaction, food restriction and ED symptoms, which may contribute to EDs [77, 78]. With the spread of social media use and network sites, this increase in the drive to thinness and body dissatisfaction could make teenagers and young adults more vulnerable to EDs, while playing a primordial role in disordered eating attitudes [79, 80]. To our knowledge, this is the first study that aimed to evaluate the mediation effect of depression and anxiety between BN and PSMU. Anxious and depressive temperaments as well as state anxiety, had a direct unmediated effect on the drive to thinness, which is a core body-related psychopathology of AN [81].

### Body dissatisfaction and BN

The results of the current study showed a positive association between body dissatisfaction and BN, which is consistent with previous findings [82]. Individuals with higher levels of body dissatisfaction usually have higher levels of abnormal eating attitudes such as drive for thinness or fear of gaining weight [83], which lead researchers to identify body dissatisfaction as a risk factor for EDs [84].

### Clinical implications

The findings of this study may help clinicians better understand the associated factors – depression, anxiety and PSMU that increase BN symptoms. They may serve as a first step to create early intervention strategies, such as Cognitive Behavior Therapy – Enhanced (CBT-E), which was proven to have an important impact on the reduction of EDs symptoms [85, 86]. As anxiety and depression were positively associated with BN, reducing their levels may in turn be associated with a decrease in BN levels; hence, other forms of treatment that tackle depression and anxiety may also be of use to reduce BN symptoms such as Cognitive Behavioral Therapy [87, 88], Schema Therapy [89], and Mindfulness-Based Interventions [90]. The need for campaigns and awareness about the harms of PSMU would also be needed in Lebanon.

### Strengths and limitations

There are some limitations in our study. The data's cross-sectional nature limits the ability to pull causality conclusions. The use of a self-administered questionnaire and the under or over-estimation of a question pose a risk for information bias. There is also a risk of selection bias, given the nature of the sample enrollment and the fact that we cannot know the refusal rate. Furthermore, a residual confounding is still possible, despite the fact that we included several factors as potential confounders. Recruitment was completed entirely online due to security and health reasons in Lebanon. Moreover, it is recommended to conduct longitudinal or cross-sectional studies taking into consideration the association between time spent on SM and other variables while taking into consideration the content consumed while using social media. Although validated in Lebanon, the SMD scale was created to screen for the possible problematic social media use in participants but not for diagnosis, since social media is not yet classified as an addiction or disorder according to the DSM-5.

Notwithstanding these limitations, the results represent preliminary evidence and could be considered as a baseline for future studies to investigate other variables associated with PSMU and BN in Lebanon. This study revealed important findings that encourage further exploration of BN and its correlates in Lebanon.

## Conclusion

BN is a serious mental and physical illness that involves complex and damaging relationships with food, eating, exercise, and body image. Improved awareness might lead to earlier detection and treatment in these groups that suffer from an extra stigma of a ‘young, Western, female-specific’ psychiatric disorder. Additional investigations of BN and its correlates must strive to improve the comprehension of these associations’ pathways through designs that allow drawing temporal frameworks, in order to efficiently treat this ED and prevent its negative outcomes. Futures studies should replicate the mediation analysis conducted in the current study, while taking into account EDs other than BN.

## Data Availability

All data generated or analyzed during this study are not publicly available due the restrictions from the ethics committee.

## Abbreviations

BN: Bulimia nervosa
PSMU: Problematic social media use
ED(s): Eating disorder(s)
AN: Anorexia nervosa
BED: Binge eating disorder
